# Strategies in Surgical Salvage: Navigating a Chest Wall Sarcoma Emergency

**DOI:** 10.7759/cureus.70424

**Published:** 2024-09-29

**Authors:** Vladimir Aleksiev, Daniel Markov, Boyko Yavorov, Spas Kitov, Kristian Bechev

**Affiliations:** 1 Cardiovascular Surgery, Medical University of Plovdiv, Plovdiv, BGR; 2 General and Clinical Pathology, Medical University of Plovdiv, Plovdiv, BGR; 3 Cardiology, Medical University of Plovdiv, Plovdiv, BGR; 4 Neurological Surgery, Universitetska Mnogoprofilna Bolnitsa za Aktivno Lechenie (UMBAL) Pulmed, Plovdiv, BGR

**Keywords:** chest wall reconstrution, myxoid pleomorphic liposarcoma, recurrence, rotational flap, sarcoma

## Abstract

Soft tissue sarcomas arising from the chest wall are an extremely rare entity. They are classified as mesenchymal tumors of varying aggression. It is this unpredictable behavior that makes them an unexpected hurdle even for experienced surgeons. Given the intricate anatomy of the thorax, any of its various tissues can potentially give rise to a sarcoma. Soft tissue sarcomas, in particular, present a diagnostic and surgical difficulty due to the necessity of complex surgical techniques and the rising need for chest wall reconstruction. These tumors are the subject of multidisciplinary discussions to come to the best therapeutic course. This is precisely what will prevent recurrence and ensure a better understanding of this pathology. In this report, we present the case of a recurrent soft tissue sarcoma that required emergency surgery due to spontaneous bleeding. It perfectly showcases the complicated treatment of such pathologies and is of profound surgical interest as it utilizes a large chest wall resection, coupled with an intricate subcutaneous flap reconstruction. In this report, we tried to outline the critical aspects of sarcoma surgery and presented our method of choice when reconstructing the soft tissues of the chest.

## Introduction

Sarcomas represent a rare group of malignant tumors, constituting about 1% of all malignancies in adults. Approximately 10-15% of sarcomas are reported to arise from the chest wall, making primary chest wall sarcoma a rare occurrence [1]. The disease shows a predilection towards the male sex, mainly affecting men in their fifth decade. A wide array of prognostic factors exists to predict the most favorable outcome for patients. These have been studied in numerous case series, such as age, sex, histology, grade, resection margins, and adjuvant treatment among others.

A recent study by Gangopadhyay et al. shows survival outlining the most frequent types of sarcomas [2]. A median follow-up of a patient cohort for 20.2 months concluded that overall two-year survival in sarcoma patients was 74.7%. Two-year overall survival (OS) and disease-free survival (DFS) of bone sarcoma were 91% and 71.3%, respectively. The Ewing sarcoma family of tumors (ESFT) exhibited a two-year OS of 50.6%, making it the most aggressive of all, while chondrosarcoma and fibromatosis demonstrated a 100% two-year OS. Out of all sarcomas, incidence rates for soft tissue, visceral and bone sarcomas tend to fall within 3.6, 2.0, and 0, 6 per 100,000 respectively. The most common histological subtypes are gastrointestinal stromal tumors (GISTs) (18%; 1.1/100,000), unclassified sarcomas (16%; 1/100,000), liposarcomas (15%; 0.9/100,000), and leiomyosarcoma (11%; 0.7/100,000). Most commonly, a soft tissue sarcoma would present as a painless mass, gradually increasing in size, which can infiltrate surrounding structures. Mass effect can occur with larger tumors, compressing nearby tissues and yielding site-specific symptoms. 

## Case presentation

**Figure 1:**
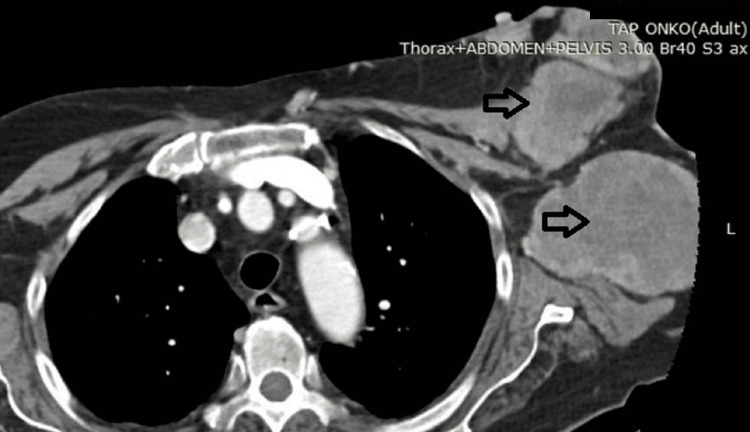
The preoperative computed tomography with the lesions marked by arrows

An 84-year-old woman was admitted to the Clinic of Thoracic Surgery after reporting an enlarging mass on her chest. Past medical history revealed an unplanned excision of a tumor on the left side of her chest, with the subsequent pathological examination confirming the diagnosis of sarcoma. Almost a year after surgery, she had noted a lump in her left breast, which had progressively grown and had now started to bleed, almost two months after she first noticed the new lesion. Upon physical examination, a 5 cm lesion was palpable in the latero-inferior quadrant of the breast, with multiple nodules around it and towards the axillae, following the previous incision. The majority of the lesions had evidence of necrosis, affecting the overlying skin, and were bleeding spontaneously. A computed tomography scan (Figure 1) revealed a total of six lesions in the soft tissues of the chest wall ranging from 3 to 14 cm in size. The patient informed us of no comorbidities, her blood work was unremarkable, and despite her age, the situation was judged as an emergency due to the several sites of severe bleeding. Thus, the patient was deemed viable for resection.

**Figure 2:**
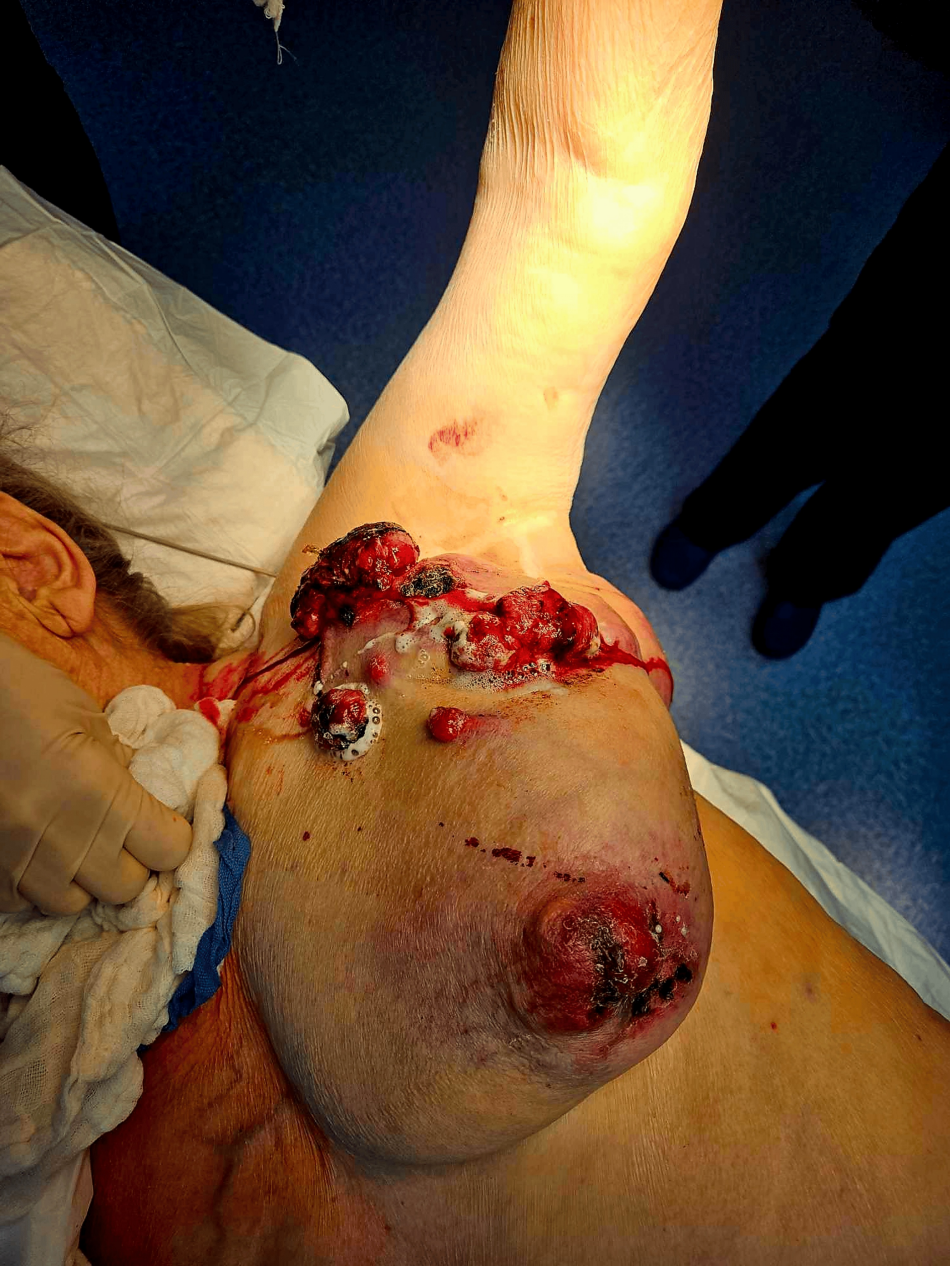
A preoperative photo, showcasing active bleeding from the neoplastic lesions

**Figure 3:**
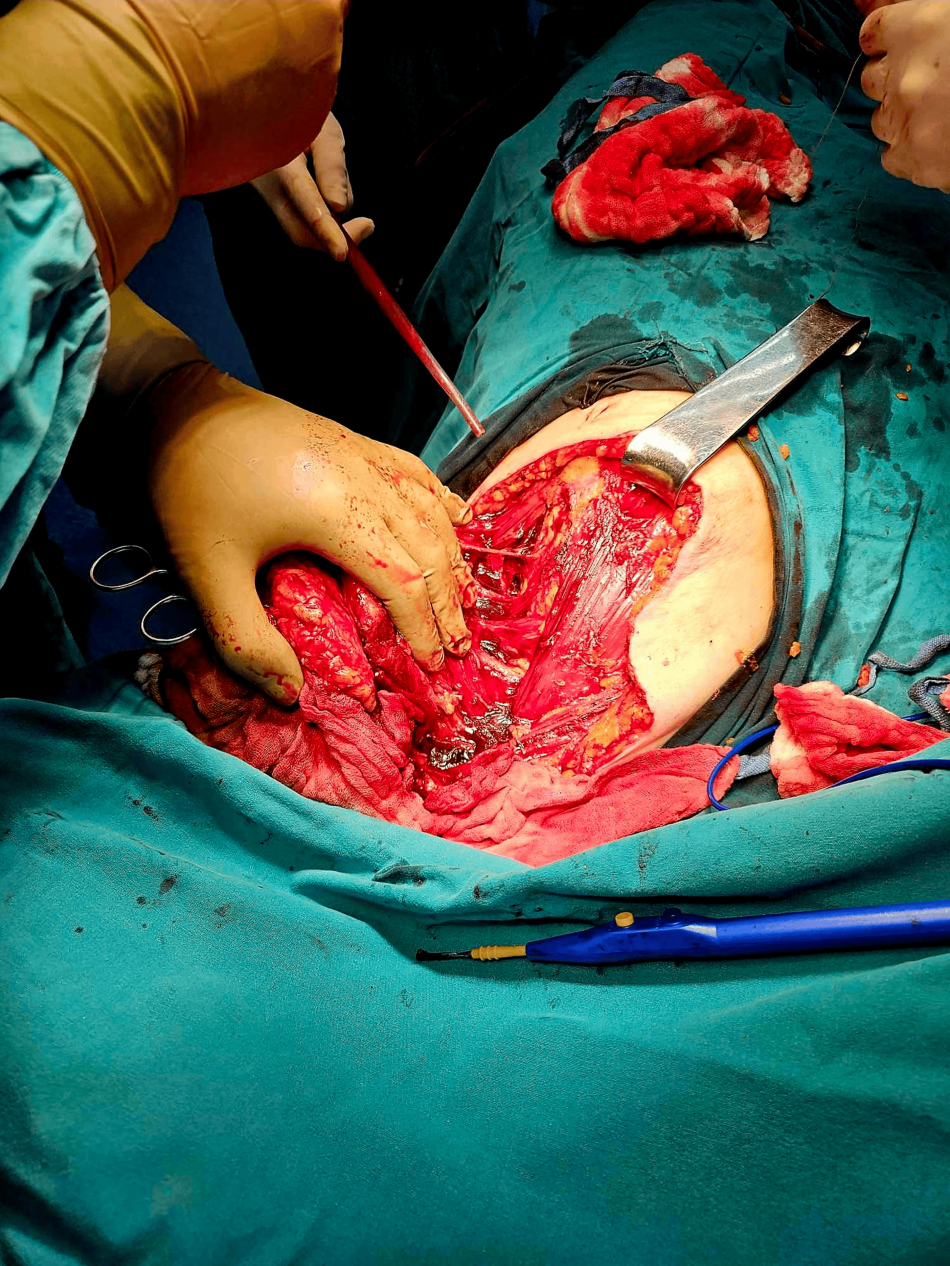
The mass being dissected free from the chest wall

**Figure 4:**
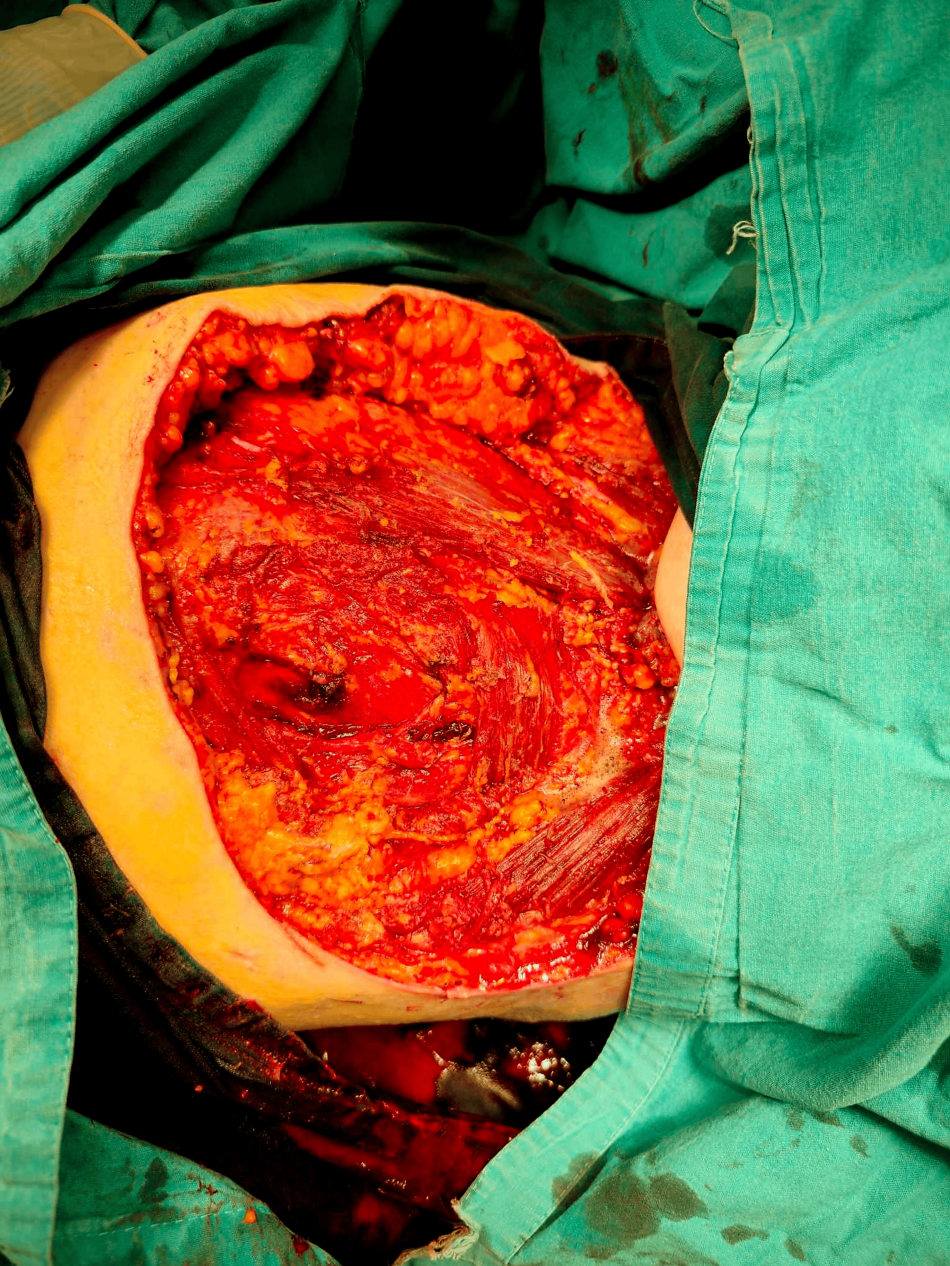
The surgical bed after resection and removal of the tumor

**Figure 5:**
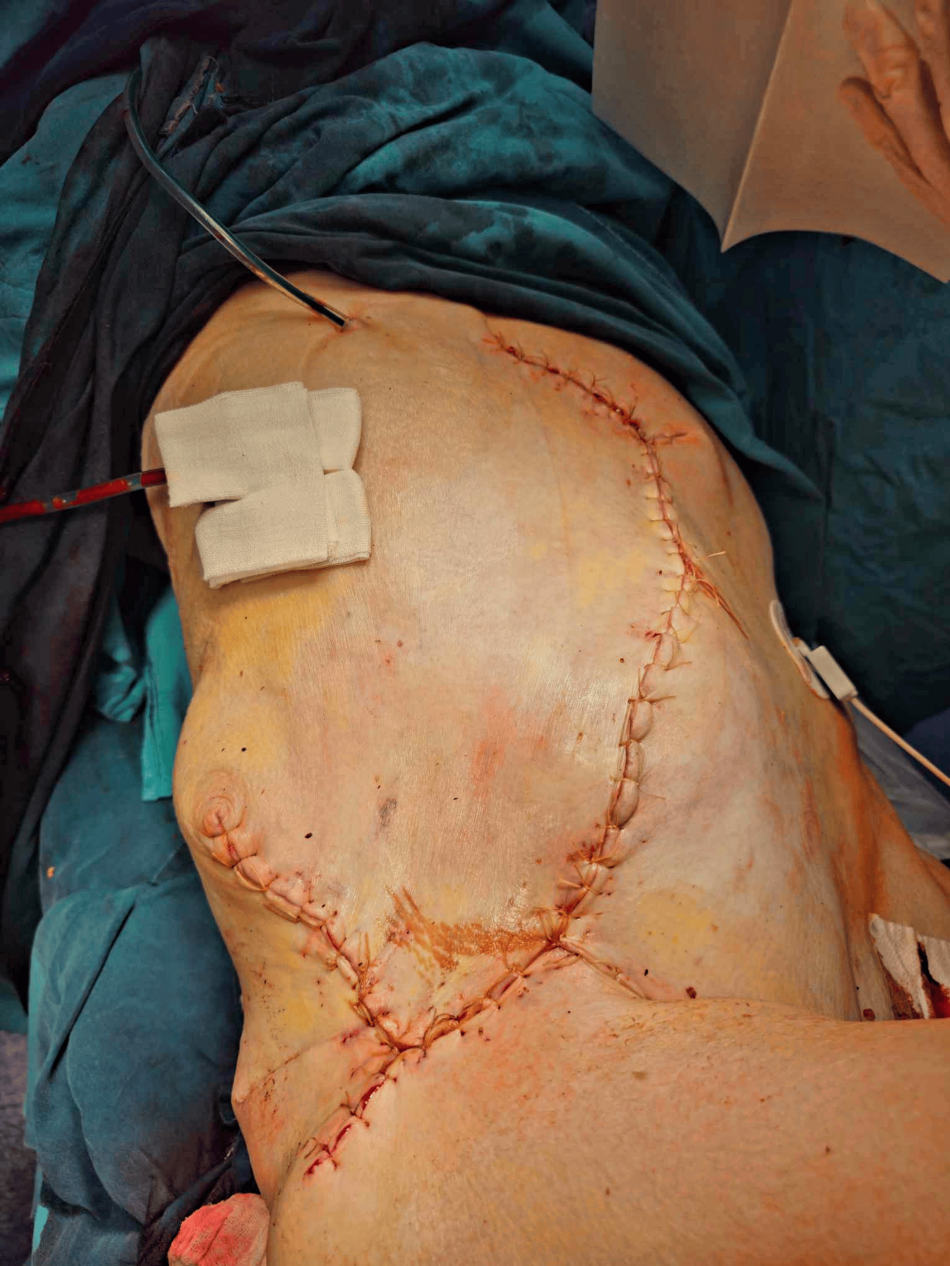
The end result after wound closure

Under general anesthesia, the patient was placed in the lateral decubitus position, ensuring the mobility of the upper extremity in the glenohumeral joint. In this way, we could freely maneuver the arm and patient in order to gain maximum exposure. All bleeding was then packed with gauze, creating a bandage that roughly outlined the skin margins. All sarcoma lesions were excised with the surrounding subcutaneous tissue, skin, and parts of the major pectoral muscle and a section of the serratus muscle, which were found to be invaded. A rotational skin flap was then created using skin from the flank, with which we covered the created defect in the chest wall (Figures 2-5). A suction drain was placed to obliterate the created cavity and prevent the accumulation of fluid.

The patient had an uneventful postoperative period up until the seventh day after surgery, when a mass started to form underneath the surgical incision. Upon revision of the wound, it was found that she had developed a recurrence, which was promptly excised. Following the second operation, the patient experienced a trouble-free recovery, and the drain was removed on the 20th day after surgery, after which she was discharged. She was then referred to the Department of Oncology and was planned to start radiation therapy. The patient reported no issues on the follow-up appointment, a full month after she underwent surgery. After discussions with a multidisciplinary Oncology team, the patient was referred for radio and chemotherapy as per protocol.

**Figure 6:**
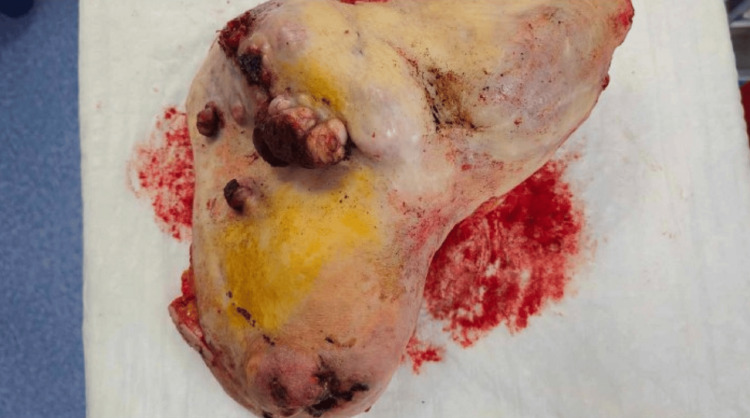
Anterior view of the specimen after resection

**Figure 7:**
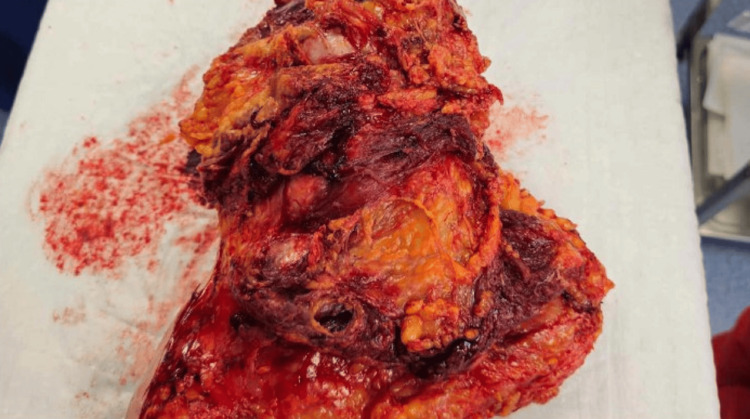
The specimen viewed from a dorsal perspective

**Figure 8:**
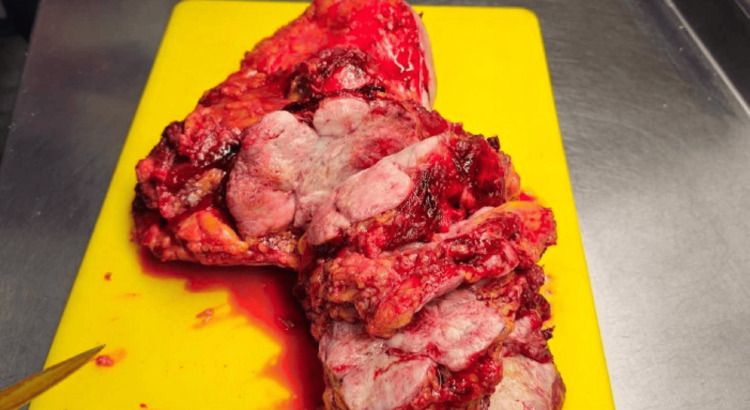
Cross-section of the specimen

On gross examination, the tumor measured 16 x 23 x 35 cm, exhibiting a greyish-white color upon sectioning (Figures 6-8). The mass had a soft consistency and lacked a well-defined capsule, allowing it to infiltrate the adjacent soft tissues. The tumor had extended into the surrounding fatty and fibrous tissues, transverse striated muscles, and the overlying skin.

Microscopic examination revealed a diffusely infiltrating soft-tissue sarcoma. The tumor was well vascularized and demonstrated significant heterogeneity, with areas composed of myxoid fields and spindle-shaped cells. In certain regions, the tumor cells were arranged in a bundle-shaped, whorled structure with numerous atypical mitoses. Atypical lipoblasts with vacuolated cytoplasm were identified, along with fields of atypical pleomorphic tumor cells, some of which were multinucleated. The histopathological findings were consistent with a diagnosis of myxoid pleomorphic liposarcoma of the soft tissues of the chest wall. Further histological analysis showed diffuse, irregularly arranged atypical spindle cells intermixed with a myxoid stroma. Importantly, there was no evidence of coagulation necrosis within the tumor.

An immunohistochemistry (IHC) study was performed to further characterize the tumor. The tumor cells demonstrated diffuse positivity for vimentin, while desmin was negative. S-100 protein was focally positive in the tumor cells, and CD34 was positive in the walls of blood vessels. The tumor cells were negative for epithelial markers (cytokeratin), mesenchymal markers (desmin, smooth muscle actin), lymphoid markers (CD3 and CD20), and melanocytic markers (HMB45). The absence of cytokeratin and HMB-45 immunoreactivity excluded the possibility of carcinoma and melanoma. The absence of CD3, CD20, and CD34 in the tumor cells ruled out diagnoses of T-cell lymphoma, B-cell lymphoma, and angiosarcoma.

**Figure 9:**
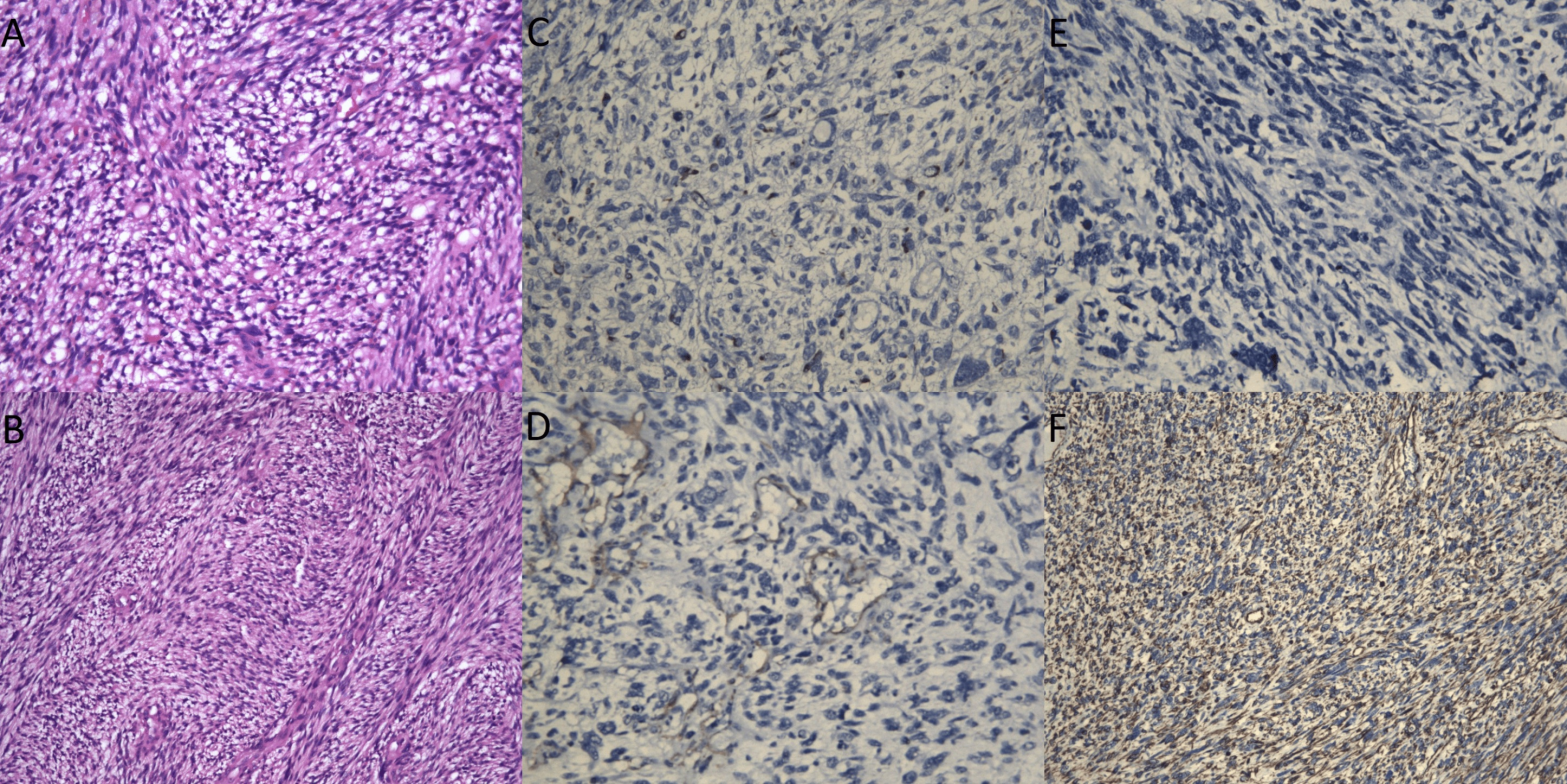
(A) Spindle-shaped pleomorphic cells with a myxoid matrix, plexiform vessels, and numerous pleomorphic lipoblasts (H&E, x100); (B) A well-vascularized malignant tumor with a vortex-like bundle structure with atypical spindle-shaped cells and lipoblasts (H&E, x40); (C) IHC testing with S-100, focal positive in tumor cells (x100); (D) IHC testing with CD 34, positive in blood vessel walls (x100); (E) IHC testing with desmin, negative in tumor cells (x100); (F) IHC testing with vimentin, diffuse positive in tumor cells (x40) IHC: immunohistochemistry; H&E: hematoxylin and eosin

The focal positivity for S-100 protein, combined with the immunopositivity for CD34, supported the diagnosis of liposarcoma. The Ki-67 proliferative index was 50%, indicating a high proliferative rate. Importantly, MDM2 gene amplification was absent (Figure 9), which is often used to differentiate liposarcoma subtypes.

The overall findings, including the macroscopic appearance, microscopic characteristics, and immunohistochemical profile, confirmed the diagnosis of myxoid pleomorphic liposarcoma of the chest wall. This case highlights the aggressive nature of this tumor, characterized by its extensive local infiltration and high proliferative activity.

## Discussion

Myxoid pleomorphic liposarcoma is a rare and aggressive subtype of liposarcoma with the most common location being in the mediastinum, followed by the head, neck, thorax, and extremities. It has a hybrid morphology, which is defined as a combination of hybrid morphological features, observed in both myxoid liposarcoma and pleomorphic liposarcoma. As evidenced by published literature, this condition is more prevalent in younger individuals, with a median age of onset below 30 years. However, it can also manifest in older patients. It is characterized by frequent recurrences, distant metastases, and a poor prognosis [3].

Myxoid pleomorphic liposarcoma is a recently defined subtype of liposarcoma. Two distinct subtypes of myxoid liposarcoma are recognized: myxoid pleomorphic liposarcoma and myxoid spindle cell liposarcoma. It is important to differentiate these subtypes from conventional myxoid liposarcoma and conventional pleomorphic liposarcoma. It is likely that myxoid pleomorphic liposarcoma and myxoid spindle cell liposarcoma represent high-grade and low-grade variants of myxoid liposarcoma, respectively.

Myxoid pleomorphic liposarcoma is histologically soft and consists of spindle-shaped pleomorphic cells, with a myxoid matrix, plexiform vessels, and numerous pleomorphic lipoblasts with increased mitotic activity [3]. This diagnosis is supported by fluorescence in situ hybridization (FISH), whole genome gene sequencing (sWGS), and DNA methylation profiling. The use of sWGS can reveal that myxoid pleomorphic liposarcomas exhibit complex chromosomal alterations, particularly the loss of 13q14, which encompasses the *RB1, RCTB2, DLEU1,* and* ITM2B* genes. Further, FISH analyses can confirm the presence of monoallelic RB1 deletion, with nuclear expression of Rb being deficient in all myxoid pleomorphic liposarcoma cases studied [4]. No *MDM2* gene amplification was demonstrated in our case. The genetic and epigenetic features of myxoid pleomorphic liposarcoma suggest a relationship with conventional pleomorphic liposarcoma, yet its distinctive clinical features warrant its separation into a new subgroup of soft tissue sarcomas.

Timely diagnosis and management of soft tissue sarcomas are associated with a favorable outcome. This process commences with a comprehensive physical examination, which is then complemented by the utilization of adequate imaging studies. While ultrasonography is a rapid and straightforward method of diagnosing tumors of the chest wall, with its B-mode ultrasonography and contrast-enhanced ultrasound (CEUS), its primary application is to obtain sufficient material for histology. A biopsy can be rapidly obtained via a tru-cut needle-automatic system, rendering the open biopsy obsolete. In order to achieve an accurate staging of the tumor, a contrast CT scan is essential, as it allows for the evaluation of the sarcoma's spread and its relationship to surrounding structures. Furthermore, MRI is employed to examine the soft tissues and bony structures of the thorax.

Local excision of the tumor presents the best prognosis for non-metastatic sarcoma. One should always strictly adhere to the sarcoma rule, which states that no tumor tissue should be seen during resection. In the case of large and highly invasive tumors, where radical resection is not possible or adequate resection margins cannot be achieved, radiation therapy is advised. In the event that the mass is not resectable from the outset, or there is a suspicion of a large tumor burden, neoadjuvant chemotherapy may be employed to enhance the likelihood of resectability.

In a retrospective review of 44 surgically treated patients with a median age of 51.8 by Tsukushi et al. from 1992 to 2006, it was found that 22 sarcomas (50%) were high-grade and 22 (50%) were low-grade [5]. A total of 31 patients (44.2%) had undergone unplanned excision prior to the study. Twenty-six patients (59.1%) were found to be continuously free of disease. The OS rate at five years was 88.5%. The local recurrence-free survival rate at five years was 88.5%. The most significant prognostic factors for recurrence are margin distance <1.5 cm and histologic grade III [5].

A study by Thomas et al. examined a total of 621 patients, 76 of whom required reconstructive surgery of the chest wall due to sarcoma [6]. The study compared 52 local/pedicled flaps with 24 free flaps. The results demonstrated that local muscle and skin, along with the free Latissimus dorsi muscle and ALT perforator flaps, were the most frequently utilized flaps. This was similar to the findings of Park et al. [7]

## Conclusions

The field of sarcoma surgery is vast and requires specific expertise and extensive insight. It is our hope that this report will serve to raise awareness of the fact that this is a tumor that requires the input of a multidisciplinary team of experts. The diagnosis and treatment plan for sarcoma are critical points that must take into account the matter of resectability and patient fitness. Finally, it is important to emphasize that this type of surgery is best performed in specialized sarcoma centers with the necessary resources and medical expertise.

We hypothesize that, in the future, tumor heterogeneity and the presence of different genetic mutations with morphological patterns will result in the separation of new subgroups of tumors, which may in turn be derived from a combination of previously classified ones.
